# Natural Occurring and Engineered Enzymes for Peptide Ligation and Cyclization

**DOI:** 10.3389/fchem.2019.00829

**Published:** 2019-11-29

**Authors:** Timo Nuijens, Ana Toplak, Marcel Schmidt, Antonio Ricci, Walter Cabri

**Affiliations:** ^1^Fresenius Kabi iPSUM, I&D Center EnzyPep B.V., Geleen, Netherlands; ^2^Fresenius Kabi iPSUM Srl, Villadose, Italy

**Keywords:** peptide, ligation, enzymatic, cyclization, conjugation

## Abstract

The renaissance of peptides as prospective therapeutics has fostered the development of novel strategies for their synthesis and modification. In this context, besides the development of new chemical peptide ligation approaches, especially the use of enzymes as a versatile tool has gained increased attention. Nowadays, due to their inherent properties such as excellent regio- and chemoselectivity, enzymes represent invaluable instruments in both academic and industrial laboratories. This mini-review focuses on natural- and engineered peptide ligases that can form a new peptide (amide) bond between the *C*-terminal carboxy and *N*-terminal amino group of a peptide and/or protein. The pro's and cons of several enzyme classes such as Sortases, Asparaginyl Endoproteases, Trypsin related enzymes and as a central focus subtilisin-derived variants are summarized. Most recent developments with regards to ligation and cyclization are highlighted.

## Introduction

Due to the increasing length and complexity of peptide pharmaceuticals, there is a growing demand for their green and efficient production (Lau and Dunn, 2018). Established methods such as recombinant expression and solid phase peptide synthesis (SPPS) have several disadvantages driving the need for new ligation and modification technologies. Using recombinant expression, it is difficult to incorporate (multiple) unnatural amino acids (AAs) or include peptide modifications such as (fatty acid) acylation or (*C*-terminal) amidation, which is much more straightforward using SPPS. However, it is still a challenge to produce longer peptides using classical SPPS due to the decrease in yield directly correlated to the peptide length. Incrementally more impurities are generated, and consequently, the purification of the final product becomes more demanding and increasingly costly. Therefore, several ligation methods have been developed to ligate smaller peptide fragments, which can be produced in higher yield and purity. Many chemical ligation methods such as native chemical (Dawson et al., 1994; Rohde and Seitz, 2010; Conibear et al., 2018; Kulkarni et al., 2018; Agouridas et al., 2019), α-Ketoacid-Hydroxylamine (Bode et al., 2006; Pusterla and Bode, 2012, 2015; Bode, 2017), Staudinger-(Maly et al., 2000; Nilsson et al., 2000; Köhn and Breinbauer, 2004), or Serine threonine ligation (Liu and Tam, 1994; Li et al., 2010; Zhang et al., 2013; Tung et al., 2015; Lee et al., 2016; Liu and Li, 2018) have become powerful tools in chemical biology, giving the access to synthetic proteins by using fragment ligation strategies. Besides chemical ligation, enzymatic ligation strategies have gained increased attention in recent years due to their inherent properties such as excellent regio- and chemoselectivity and the catalysis of reactions under mild conditions (Schmidt et al., 2017b; Nuijens and Schmidt, 2019). The variety of enzymes used for enzymatic ligation mainly includes proteases and engineered variants thereof as well as transpeptidases. Even though proteases are very abundant in nature, few enzymes, namely ligases, have been found that naturally catalyze the reverse reaction, i.e., peptide bond formation. Triggered by this, researches have started exploiting and engineering proteases to act as ligases (Jakubke, 1995). In this mini-review, we describe the currently existing set of ligases and recent developments, both for intermolecular and intramolecular (cyclization) ligation. Here, we consider only enzymes that catalyze the formation of a native peptide bond. Roughly four classes of peptide ligases are discovered up to date, i.e., Sortases, Asparaginyl Endoproteases, Trypsin related enzymes and subtilisin-derived variants. The main focus will be on the recent rise and applications of subtilisin-type of enzymes.

## Sortases

In nature, Sortase A from *Staphylococcus aureus* catalyzes the covalent anchoring of surface proteins to the cell wall (Marraffini et al., 2006). First, it cleaves off the *C*-terminal glycine of an LPXTG recognition motif (X = any amino acid) and couples the threonyl carboxylate to the *N*-terminal amino group of a pentaglycine peptide attached to peptidoglycan (Figure 1.1A). This transpeptidation reaction by Sortase A has been applied as a synthetic tool for peptide and protein conjugation (Schmidt et al., 2017b) as well as for peptide and protein (*C*-to-*N*, i.e., head-to-tail) cyclization (Antos et al., 2009; Wu et al., 2011; van't Hof et al., 2015). During catalysis the motif R_1_-LPXT-*G-R*_2_ (R_1_,R_2_,,R_3_ = proteins, synthetic peptides, solid supports or cells) is recognized and the Thr-Gly amide bond is cleaved by the active site thiol to form an acyl-enzyme (thioester) complex with glycine as leaving group. The thioester complex is resolved by nucleophilic attack of a peptide with an *N-*terminal glycine (GR_3_), yielding a native R_1_-LPXT-*G-R*_3_ peptide bond, see Figure 1.1C for ligation examples.

**Figure 1:**
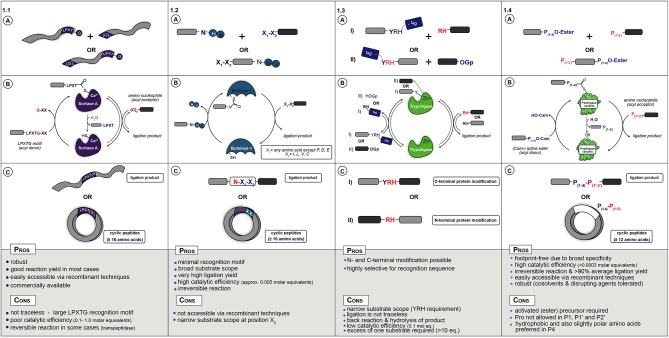
**(A)** Starting materials for enzymatic ligation using Sortase **(1.1)**, Butelase **(1.2)**, Trypsiligase **(1.3)**, and Peptiligase **(1.4)** variants. **(B)** Catalytic mechanism **(C)** ligation products.

Sortase A is a very robust enzyme that can be produced recombinantly in moderate yields (>40 mg/L) and is commercially available. The peptide starting materials are also easily accessible either via conventional synthetic or recombinant strategies. Nowadays, the *sortagging* reaction has been adopted for a wide range of applications such as protein ligation (Policarpo et al., 2014), peptide fusion (Agwa et al., 2018), *N-* and *C-*terminal labeling of proteins and antibodies (Beerli et al., 2015; Chen et al., 2016), cell-surface modification (Swee et al., 2015), protein immobilization (Ito et al., 2010), or peptide cyclization (Jia et al., 2014). Already in 2004 sortagging was described for protein/peptide ligation to another protein/peptide, even containing unnatural AAs (Mao et al., 2004). This was further extended to the coupling of fluorescent-labels or -proteins to proteins of interest (Matsumoto et al., 2012, 2016; Ott et al., 2016) and the cross linking of enzymes (Li et al., 2017) or ligation of two protein domains (Omura et al., 2018; Raltchev et al., 2018). Peptide ligation to proteins has, among others, been exemplified by coupling peptides containing thioesters (Ling et al., 2012), cell penetrating ability (Van Lith et al., 2017), non-canonical AAs (Ke et al., 2017), antimicrobial activity (Touti et al., 2018), purification tags (Bellucci et al., 2013), isotopes for labeling (e.g., NMR) (Freiburger et al., 2015; Williams et al., 2016; Sonntag et al., 2017), or ligation handles such as azides (Ta et al., 2018). More recent Sortase-catalyzed peptide ligation examples include the synthesis of relaxin analogs (Wang et al., 2018), spider venom peptides (Agwa et al., 2018), and labeled peptides with e.g., lipids, biotin or PEG (Cheng et al., 2017). Cyclization (Figure 1.1B) has been shown for the cyclotide kalata B (Jia et al., 2014), for sunflower Trypsin inhibitor SFT-1 (Zhang et al., 2015), for cyclotide MCoTI-II via recombinant expression coupled to Sortase A-mediated backbone cyclization (Stanger et al., 2014), and for the synthesis of cyclic analogs of the antibacterial peptide P-113 (Wu et al., 2017) and salivary peptide histatin (Bolscher et al., 2011). In general, it could be shown that (glyco-)peptides with 16 or more AAs could be cyclised with good efficiency (>80% conversion). Besides peptides, proteins such as the green fluorescent protein (Parthasarathy et al., 2007; van't Hof et al., 2015) or cytokines (Popp et al., 2011) can also be successfully cyclised in moderate to excellent yields (Rasche et al., 2016).

The main drawbacks of Sortases are the strict sequence requirements, which remain present in the ligation product, the poor catalytic efficiency and reversibility of the reaction leading to low yield and product hydrolysis. To expand *sortagging* beyond the standard LPXT-*G* motif, Sortase homologs as well as engineered variants have been reported, although with limited success (Dorr et al., 2014; Antos et al., 2016; Nikghalb et al., 2018). Besides the substrate scope, Sortase variants with increased thermal and chemical stability (Pelay-Gimeno et al., 2018) or activity (Beerli et al., 2015) have been described. Another method to circumvent the poor reaction kinetics is via proximity-based Sortase-mediated ligation (PBSL), which enables ligation efficiencies of over 95%. For PBSL the target protein and sortase are linked using the SpyTag-SpyCatcher protein pair. Although after ligation the Spytag is cleaved off and the target protein is released, this approach requires elaborate reaction engineering and Spycatcher modified and His_6_-tagged sortase is required in equimolar amounts (Wang et al., 2017). Besides protein engineering, another successful strategy used is reactant engineering that renders the transpeptidation reaction irreversible. One approach uses modified depsipeptide substrates that upon transpeptidation release non-reactive fragments, e.g., a non-reactive hydroxyacetate moiety (Williamson et al., 2012, 2014) or spontaneously form a diketopiperazine (Liu et al., 2014).

In conclusion, when addition of the sorting sequence LPXTG to a peptide or protein does not interfere with its function, *sortagging* represents a powerful tool for site-selective bioconjugation (Figure 1.1C). Nevertheless, its broad application is still hampered by the low catalytic efficiency (large quantity of enzyme required), long reaction times, moderate yields and the high molar equivalents of one of the substrates needed to drive the equilibrium toward product. Despite the shortcomings, mainly due to easy accessibility of enzyme and substrates, sortagging has become a popular tool in chemical biology.

## Asparaginyl Endoproteases

More recently discovered and a promising alternative to Sortases is the application of asparaginyl endoproteases (AEP) such as Butelase 1 (Nguyen et al., 2014; James et al., 2017; Jackson et al., 2018). Butelase 1, isolated from the tropical plant (*Clitoria ternatea)* is an Asx-specific (Asx = Asn or Asp) cysteine transpeptidase that natively catalyzes peptide head-to-tail cyclization in the biosynthesis of cyclotides (Craik et al., 1999). As with Sortase, AEP enzymes cleave a recognition sequence, in this case N-HV or D-HV, to form a thioester acyl-enzyme intermediate that is resolved by nucleophilic attack by a peptide *N*-terminal amine (Figure 1.2A). A major advantage is the relatively short recognition sequence, the His-Val motif is cleaved off and only an Asx residue is left as a footprint at the ligation site.

Butelase 1 has a broad tolerance for the first (*N*-terminal) residue to be coupled (any AA except Pro, Asp, and Glu), but at the second position Ile, Leu, Val, or Cys is required (Nguyen et al., 2016a). Compared to Sortases, Butelase 1 features substantially higher catalytic efficiency (only ~0.005 molar equivalents of enzyme required). The peptide substrates containing the Asx-His-Val motif can be easily prepared via straightforward SPPS or recombinant expression. Butelase 1 has been shown to efficiently promote intermolecular peptide ligation as well as head-to-tail macrocyclization of peptides from 10 residues or longer in nearly quantitative yields (Figure 1.2B) (Nguyen et al., 2014, 2015b, 2016b; Hemu et al., 2016). As in nature, it preferably catalyzes cyclization over hydrolysis. For example, kalata B1, GFP, and human growth hormone (somatropin) were cyclised with excellent efficiency (>95% yield) (Nguyen et al., 2015b). Furthermore, Tam and coworkers recently reported the first chemical synthesis of large circular bacteriocins such as the 70-mers AS-48 and uberolysin (Hemu et al., 2016). Interestingly, Butelase 1 has the ability to cyclise peptides consisting of almost exclusively d-AAs, except for the *C-*terminal Asx residue (Nguyen et al., 2016a). Besides cyclization, Butelase-1 can be used for the modification of live cell bacterial surfaces (Bi et al., 2017), for the semi-synthesis of ubiquitin (Nguyen et al., 2015a), and to prepare large circular bacteriocins, the largest antimicrobial peptides known up to date (Hemu et al., 2016). Other possibilities are the preparation of peptide dendrimers using lysine derived scaffolds (Cao et al., 2016) or even the modification of proteins (Nguyen et al., 2015a). For example, Ploegh et al. described a one-pot dual labeling approach for the sequential modification of heterodimeric proteins such as antibodies with different labels at light and heavy chain, respectively, as well as an approach for the sequential *C-*to*-C* fusion of two protein of interest (Harmand et al., 2018). Butelase-1 can also be applied in the synthesis of protein *C*-terminal thioesters and thus enabling tandem chemoenzymatic ligations (e.g., via NCL) (Liu et al., 2015). Butelase prefers intra- over inter-molecular ligations for which a large excess of nucleophile is required and the N-terminus of the acyl donor should be protected or outside the Butelase substrate scope.

As glycine for Sortases, the cleaved HV-dipeptide by Butelase acts as a competitive nucleophile with the substrate of interest, therefore requiring a huge access of reactant. To overcome this limitation, the use of thiodepsipeptide substrates is successful in rendering the reaction irreversible (Nguyen et al., 2015a). However, this strategy involves the use of unstable thioester substrates and does not prevent hydrolysis of the product. Besides the drawbacks of sequence specificity (Asx footprint), hydrolytic activity and reversibility, Butelase-1 has to be isolated from plants, therefore limiting its potential in biotechnological applications. So far, recombinant expression has not been successful, although this will probably be achieved in the near future. Recently, a markedly less active AEP named OaAEP1, has been recombinantly expressed in *Escherichia coli*. Although titers were low (<2 mg/L), OaAEP1 has the advantage of being a fully characterized enzyme that is able to cyclise a diverse range of substrates (~90 times slower than Butelase 1) (Harris et al., 2015; Yang et al., 2017). Later studies showed that the catalytic efficiency of native OaAEP1 could be improved through structure-based enzyme engineering (Yang et al., 2017), however, Butelase 1 is still most often the enzyme of choice (Yang et al., 2017).

Clearly, Butelase type enzymes have some advantages over Sortases such as minimal recognition motif, broader substrate scope and much higher catalytic activity (Figure 1.2C). However, poor accessibility of the enzymes has so far limited its application.

## Trypsin Related Enzymes

The use of native Trypsin and engineered variants for peptide synthesis has been known for decades (Nuijens et al., 2012). Recently, Bordusa et al. discovered a new engineered Trypsin variant, termed Trypsiligase, which can be used for the *N-* and *C-*terminal modification of protein or peptide substrates (Liebscher et al., 2014b). Trypsiligase adopts an inactive partially disordered zymogen-like conformation and represents a striking example for substrate-activated catalysis, as it is exclusively active in the presence of a YRH tripeptide motif and Zn^2+^ ions (Liebscher et al., 2014b). Trypsiligase can be used for the efficient labeling of proteins bearing an *N*-terminal RH motif, which proceed via the use of activated substrates (such as peptidyl 4-guanidinophenyl esters) as acyl donors (Figure 1.3A) (Meyer et al., 2016). *C-*terminal modification can be achieved by a transpeptidation reaction between a peptide- or protein-Y-RH recognition sequence and a RH-X (X = peptide, tag) nucleophilic acyl acceptor peptide (Figure 1.3B) (Liebscher et al., 2014a). The ligation reaction is usually complete within minutes and requires ~0.1 molar equivalents of enzyme with an excess of the corresponding acyl acceptor substrate (often 10 eq.) because it needs to compete with the RH leaving group.

The Y-RH recognition motif is rare and only found in 0.5% of all known protein sequences (Liebscher et al., 2014b). Therefore, the use of Trypsiligase is restricted with regards to synthesis of native peptides and proteins, similarly to Sortase A (Figure 1.3C). Another drawback is the presence of the Y-RH sequence in the ligation product (*C*-terminal protein modification) leading to a reversible reaction and undesired hydrolysis.

## Subtilisin-Derived Variants

Ligases from nature such as Sortase and Butelase rely on a cysteine residue in the active site that forms a thioester with the acyl-donor peptide. Over 50 years ago the active site serine of a subtilisin protease was chemically converted to cysteine. Although this enzyme had an increased acylation (ligase) over hydrolysis (protease) rate, the enzyme activity was extremely low (Polgár and Bender, 1967). A few decades later, Wells et al. discovered that one additional mutation was required to reduce the steric crowding created by the thiol residue to restore the enzyme activity. This double mutant of a serine protease from *Bacillus amyloliquefaciens*, i.e., subtilisin BPN′, was termed Subtiligase (Braisted et al., 1997; Weeks and Wells, 2019). Although this mutant exhibits considerable ligase activity it still lacks satisfactory efficacy, as a huge excess of the acyl acceptor fragment is required to suppress substantial amounts of hydrolysis. However, its ligase specificity has recently been engineered in a proteome-wide screening approach, enabling the *N*-terminal labeling of diverse proteins (Weeks and Wells, 2017). During the past 10 years there has been a revival of the subtilisin based peptide ligases by the discovery of a novel Ca^2+^-independent and stable variant, termed Peptiligase (Toplak et al., 2016). This variant efficiently catalyzes peptide bond formation between a *C-*terminal ester [preferably carboxyamidometyl ester (Nuijens et al., 2016b), Figure 1.4A] fragment and an acyl-acceptor nucleophile with, in many cases, insignificant amounts of hydrolysis. Since the ester to amide conversion via a thioester intermediate (acyl-enzyme complex) is virtually irreversible, a theoretical quantitative yield of 100% can be achieved using a one-to-one molar ratio of the substrates. Peptiligase has a very high catalytic efficiency (<0.0003 molar equivalents required) and the enzyme can be easily obtained from *Bacillus subtilis* (>0.5 g/ L) (Pawlas et al., 2019). The ligation reaction of unprotected peptide fragments proceeds in aqueous media (neutral to slightly basic pH) at ambient temperature with extremely high average ligation yields (up to 98% in <1 h). Only a low molar excess of acyl acceptor (1.1–2 molar equivalents) is required (Schmidt et al., 2017b). Compared to other peptide ligases, Peptiligase is exceptionally thermostable (T_M_ = 66°C) and tolerates the presence of organic co-solvents [e.g., up to 50% (v/v) dimethylformamide (DMF)] and disrupting agents (e.g., 2 M urea or guanidinium chloride), therefore also enabling the ligation of poorly soluble or folded peptides (Toplak et al., 2016).

Peptiligase has six distinct substrate recognition pockets: four recognizing the *C-*terminal part of the peptide (S1-S4), and two involved in binding the *N-*terminal acyl acceptor part of the peptide (S1′ and S2′). After it's discovery, it was found that especially the S1' pocket was highly discriminating, only able to accommodate small AAs such as Gly, Ser, and Ala. However, using computational design and site-directed engineering, the substrate scope of this pocket could be radically broadened (Nuijens et al., 2016a). Several years of engineering focused on ligation efficiency and broad substrate scope resulted in the discovery of Omniligase-1 (Nuijens et al., 2016c). This enzyme provides an excellent basis for efficient and completely footprint-free inter- and intramolecular peptide ligation for almost any peptide sequence. For instance, it was shown that Omniligase-1 could be applied for the synthesis of the 39-mer pharmaceutical peptide exenatide in excellent yield (Pawlas et al., 2019). Most importantly, it was later shown that the enzymatic ligation technology using Omniligase-1 is scalable and robust enough for industrial application (Nuijens et al., 2016b). Exenatide was prepared at >100 gram scale with a quantified ligation yield of 88% using crude fragments made by chemical synthesis. The overall yield proved almost twice as high compared to established solid phase productions methods and the product was obtained within pharmacopeia specifications. Besides exenatide, it was shown that ligation to proteins or polymers, such as human serum albumin or the polymer XTEN is also possible. Using 4 equivalents of ester, over 95% *N*-terminal ligation efficiency leading to products >500 AAs could be achieved (Nuijens, 2016). Finally, besides peptides and conjugates, Omniligase-1 has been applied for the head-to-tail cyclization of peptides. Peptides over 12 AAs, even when containing isopeptide bonds, polyethylene glycol or d-AAs in the sequence, were cyclised in over 95% efficiency (Schmidt et al., 2017a). In the same article, the one pot synthesis and folding of the natural occurring cyclotide MCoTI-II at multi gram scale was described as well as the combination of Omniligase-1 catalyzed cyclization with chemical rigidification using tris(bromomethyl)benzene. Later, other disulfide rich peptides such as kalata B1 and RTD-1 were synthesized and successfully folded to their native conformations (Schmidt et al., 2019). It was shown that due to the broad substrate scope and traceless ligation, different sites could be used to synthesize the cyclic peptides. Most recently, the cyclization technology was combined with small organic scaffolds and other ligation technologies such as oxime ligation and click chemistry (Richelle et al., 2018; Streefkerk et al., 2019). Combining enzymatic and chemical ligation technologies, tetracyclic peptides could be synthesized in a one pot fashion that poses two distinct biological activities.

In addition to Peptiligase variants with a broad substrate scope such as Omniligase-1, enzyme engineering efforts also yielded Peptiligase variants with redesigned substrate profiles that allow selective peptide couplings. For instance, variants that can discriminate between small and large side-chains, hydrophobic and polar or negative vs. positive charge. One example is the development of a Peptiligase variant for the synthesis of Thymosin-alpha-1, termed Thymoligase. This enzyme has a preference for a positively charged AA in P1 (Lys or Arg) and a negatively charged AA in P1′ (Asp or Glu) (Schmidt et al., 2018). Two crude 14-mer peptides could be ligated in high efficiency to make the 28-mer product, which could be isolated in >98% purity after one single preparative HPLC step. Besides peptides, the substrate specific ligases could also be used for the selective coupling to heterodimeric proteins, such as the heavy/light chain of antibodies or A- and B-chain of insulin (Nuijens, 2016). Antibodies with two different tags could be prepared with almost quantitative ligation efficiency and heavy vs. light chain selectivity. A summary of possible peptide ligation and cyclization reactions is illustrated in Figure 1.4B.

In addition to using esters, thioester substrates have recently been described as more efficient substrates for Subtiligase-catalyzed ligation, drastically broadening the substrate scope (Tan et al., 2018). Moreover, sequential enzymatic ligations (coupling followed by activation) could be performed, e.g., using peptide hydrazides (Fang et al., 2011; Flood et al., 2018) or enzyme-catalyzed expressed protein ligation (Henager et al., 2016).

In conclusion, both Peptiligase and Subtiligase variants represent valuable tools in peptide-peptide ligation, as well as for the site-specific modification of proteins (Figure 1.4C) (Schmidt, 2019). In particular, Peptiligase variants such as Omniligase-1 have the potential to establish as the preferred method for the synthesis of long (pharmaceutical) peptides and protein-conjugates in a cost-efficient and environmentally friendly approach. Peptiligase-mediated coupling is scalable and can be used either as a versatile stand-alone technology or as an addition to chemical ligation methodologies (e.g., NCL) or intein-based protein ligation in both academic research labs and industrial settings.

## Recommendations

Clearly, a diverse set of ligases is available for peptide ligation and cyclization, all with their own specific advantages and disadvantages. When an enzyme recognition motif in the ligation product is not an issue and one of the reactants can be used in high excess, Sortase mediated ligation is most straight forward. The enzyme is efficient and easily accessible to any laboratory. For peptide cyclization's Butelase is one of the most efficient enzymes known, although application could be challenging because the enzyme is hard to obtain. It is less efficient for intramolecular ligations since a large excess of one of the reactants is required and protecting groups might be needed. For protein labeling, Trypsiligase is a highly selective ligase, with a very specific recognition motif. Because of the similarities to Sortase (recognition motif, transpeptidation, excess of one reactant), the latter is more often applied simply because it is commercially available. For traceless ligation and cyclization of peptides, Peptiligases are the best option. There is no need for a large excess of the reagents and the enzymes are easy to produce. However, an active ester starting material is required, which is relatively easy for peptides but not straight forward for proteins.

## Author Contributions

All authors contributed to the writing of this mini review.

### Conflict of Interest

TN, AT, and MS were employed by the company EnzyPep B.V. AR and WC were employed by the company Fresenius Kabi iPSUM Srl.

## References

[B1] Agouridas V. Mahdi O. E. Diemer V. Cargoët M. Monbaliu J. C. M. Melnyk O. (2019). Native chemical ligation and extended methods: mechanisms, catalysis, scope, and limitations. Chem. Rev. 119, 7328–7443. 10.1021/acs.chemrev.8b00712 31050890

[B2] Agwa A. J. Blomster L. V. Craik D. J. King G. F. Schroeder C. I. (2018). Efficient enzymatic ligation of inhibitor cystine knot spider venom peptides. Bioconjug. Chem. 29, 3309–3319. 10.1021/acs.bioconjchem.8b00505 30148615

[B3] Antos J. M. Popp M. W. L. Ernst R. Chew G. L. Spooner E. Ploegh H. L. (2009). A straight path to circular proteins. J. Biol. Chem. 284, 16028–16036. 10.1074/jbc.M901752200 19359246 PMC2708896

[B4] Antos J. M. Truttmann M. C. Ploegh H. L. (2016). Recent advances in sortase-catalyzed ligation methodology. Curr. Opin. Struct. Biol. 38, 111–118. 10.1016/j.sbi.2016.05.021 27318815 PMC5010448

[B5] Beerli R. R. Hell T. Merkel A. S. Grawunder U. (2015). Sortase enzyme-mediated generation of site-specifically conjugated antibody drug conjugates with high *in vitro* and *in vivo* potency. PLoS ONE 10:e0131177. 10.1371/journal.pone.0131177 26132162 PMC4488448

[B6] Bellucci J. J. Amiram M. Bhattacharyya J. McCafferty D. Chilkoti A. (2013). Three-in-one chromatography-free purification, tag removal, and site-specific modification of recombinant fusion proteins using Sortase A and elastin-like polypeptides. Angew. Chem. Int. Ed. 52, 3703 −3708. 10.1002/anie.201208292 23424160 PMC3723126

[B7] Bi X. Yin J. Nguyen G. K. T. Rao C. Halim N. B. A. Hemu X.. (2017). Enzymatic engineering of live bacterial cell surfaces using butelase 1. Angew. Chem. Int. Ed. 56, 7822–7825. 10.1002/anie.201703317 28524544

[B8] Bode J. W. (2017). Chemical protein synthesis with the α-ketoacid-hydroxylamine ligation. Acc. Chem. Res. 50, 2104–2115 10.1021/acs.accounts.7b00277 28849903

[B9] Bode J. W. Fox R. M. Baucom K. D. (2006). Chemoselective amide ligations by decarboxylative condensations of N-alkylhydroxylamines and alpha-ketoacids. Angew. Chem. Int. Ed. 45, 1248–1252. 10.1002/anie.200503991 16416482

[B10] Bolscher J. G. M. Oudhoff M. J. Nazmi K. Antos J. M. Guimaraes C. P. Spooner E.. (2011). Sortase A as a tool for high-yield histatin cyclization. FASEB J. 25, 2650–2658. 10.1096/fj.11-182212 21525488

[B11] Braisted A. C. Judice J. K. Wells J. A. (1997). Synthesis of proteins by subtiligase. Methods Enzymol. 289, 298–313. 10.1016/S0076-6879(97)89053-2 9353727

[B12] Cao Y. Nguyen G. K. T. Chuah S. Tam J. P. Liu C. F. (2016). Advances in site-specific and linkage-specific ligation. Bioconjug. Chem. 27, 2592–2596. 10.1021/acs.bioconjchem.6b00538 27723303

[B13] Chen L. Cohen J. Song X. Zhao A. Ye Z. Feulner C. J.. (2016). Improved variants of SrtA for site-specific conjugation on antibodies and proteins with high efficiency. Sci. Rep. 6:31889. 10.1038/srep31899 27534437 PMC4989145

[B14] Cheng X. Zhu T. Hong H. Zhou Z. Wu Z. (2017). Sortase A-mediated on-resin peptide cleavage and in situ ligation: an efficient one-pot strategy for the synthesis of functional peptides and proteins. Org. Chem. Front. 4, 2058 −2062. 10.1039/C7QO00481H

[B15] Conibear A. C. Watson E. E. Payne R. J. Becker C. F. W. (2018). Native chemical ligation in protein synthesis and semi-synthesis. Chem. Soc. Rev. 47, 9046–9068. 10.1039/C8CS00573G 30418441

[B16] Craik D. J. Daly N. L. Bond T. Waine C. (1999). Plant cyclotides: a unique family of cyclic and knotted proteins that defines the cyclic cystine knot structural motif. J. Mol. Biol. 294, 1327–1336. 10.1006/jmbi.1999.3383 10600388

[B17] Dawson P. E. Muir T. W. Clark-Lewis I. Kent S. B. (1994). Synthesis of proteins by native chemical ligation. Science 266, 776–779. 10.1126/science.7973629 7973629

[B18] Dorr B. M. Ham H. O. An C. Chaikof E. L. Liu D. R. (2014). Reprogramming the specificity of sortase enzymes. *Proc. Natl. Acad. Sci*. U.S.A. 111, 13343–13348. 10.1073/pnas.1411179111 PMC4169943 25187567

[B19] Fang G. M. Li Y. M. Shen F. Huang Y. C. Bin Li J. Lin Y.. (2011). Protein chemical synthesis by ligation of peptide hydrazides. Angew. Chem. Int. Ed. 50, 7645–7649. 10.1002/anie.201100996 21648030

[B20] Flood D. T. Hintzen J. C. J. Bird M. J. Cistrone P. A. Chen J. S. Philip E. (2018). Leveraging the knorr pyrazole synthesis for the facile generation of thioester surrogates for use in NCL. Angew. Chem. Int. Ed. 57, 11634–11639. 10.1002/anie.201805191 PMC6126375 29908104

[B21] Freiburger L. Sonntag M. Hennig J. Li J. Zou P. Sattler M. (2015). Efficient segmental isotope labeling of multi-domain proteins using Sortase A. *J. Biomol*. NMR 63, 1–8. 10.1007/s10858-015-9981-0 26319988

[B22] Harmand T. J. Bousbaine D. Chan A. Zhang X. Liu D. R. Tam J. P.. (2018). One-pot dual labeling of IgG 1 and preparation of C-to-C fusion proteins through a combination of Sortase A and butelase 1. Bioconjug. Chem. 29, 3245–3249. 10.1021/acs.bioconjchem.8b00563 30231608 PMC6429940

[B23] Harris K. S. Durek T. Kaas Q. Poth A. G. Gilding E. K. Conlan B. F.. (2015). Efficient backbone cyclization of linear peptides by a recombinant asparaginyl endopeptidase. Nat. Commun. 6:10199. 10.1038/ncomms10199 26680698 PMC4703859

[B24] Hemu X. Qiu Y. Nguyen G. K. T. Tam J. P. (2016). Total synthesis of circular bacteriocins by butelase 1. J. Am. Chem. Soc. 138, 6968–6971. 10.1021/jacs.6b04310 27206099

[B25] Henager S. H. Chu N. Chen Z. Bolduc D. Dempsey D. R. Hwang Y.. (2016). Enzyme-catalyzed expressed protein ligation. Nat. Methods 13, 925–927. 10.1038/nmeth.4004 27669326 PMC5088058

[B26] Ito T. Sadamoto R. Naruchi K. Togame H. Takemoto H. Kondo H.. (2010). Highly oriented recombinant glycosyltransferases: site-specific immobilization of unstable membrane proteins by using staphylococcus aureus Sortase A. Biochemistry 49, 2604–2614. 10.1021/bi100094g 20178374

[B27] Jackson M. A. Gilding E. K. Shafee T. Harris K. S. Kaas Q. Poon S.. (2018). Molecular basis for the production of cyclic peptides by plant asparaginyl endopeptidases. Nat. Commun. 9:2411 10.1038/s41467-018-04669-9 29925835 PMC6010433

[B28] Jakubke H. -D. (1995). English peptide ligases—tools for peptide synthesis. Angew. Chemie Int. Ed. 34, 175–177. 10.1002/anie.199501751

[B29] James A. M. Haywood J. Mylne J. S. (2017). Macrocyclization by asparaginyl endopeptidases. New Phytol. 218, 923–928. 10.1111/nph.14511 28322452

[B30] Jia X. Kwon S. Wang C.-I. Huang Y.-H. Chan L. Y. Tan C. C. (2014). Semienzymatic cyclization of disulfide-rich peptides using Sortase A. J. Biol. Chem. 289, 6627–6638. 10.1074/jbc.M113.539262 24425873 PMC3945325

[B31] Ke H. Matsumoto S. Murashima Y. Taniguchi-tamura H. Miyamoto R. Yoshikawa Y.. (2017). Structural basis for intramolecular interaction of post-translationally modified H-Ras∙GTP prepared by protein ligation. FEBS Lett. 591, 2470–2481. 10.1002/1873-3468.12759 28730604

[B32] Köhn M. Breinbauer R. (2004). The Staudinger ligation-a gift to chemical biology. Angew. Chem. Int. Ed. 43, 3106–3116. 10.1002/anie.200401744 15199557

[B33] Kulkarni S. S. Sayers J. Premdjee B. Payne R. J. (2018). Rapid and efficient protein synthesis through expansion of the native chemical ligation concept. Nat. Rev. Chem. 2:122 10.1038/s41570-018-0122

[B34] Lau J. L. Dunn M. K. (2018). Therapeutic peptides: Historical perspectives, current development trends, and future directions. Bioorg. Med. Chem. 26, 2700–2707. 10.1016/j.bmc.2017.06.052 28720325

[B35] Lee C. L. Liu H. Wong C. T. T. Chow Y. Li X. (2016). Enabling N-to-C Ser/Thr ligation for convergent protein synthesis via combining chemical ligation approaches. J. Am. Chem. Soc. 138, 10477–10484. 10.1021/jacs.6b04238 27479006

[B36] Li K. Zhang R. Xu Y. Wu Z. Li J. Zhou X. (2017). Sortase A-mediated crosslinked short-chain dehydrogenases/reductases as novel biocatalysts with improved thermostability and catalytic efficiency. Sci. Rep. 7:3081. 10.1038/s41598-017-03168-z 28596548 PMC5465079

[B37] Li X. Lam H. Y. Zhang Y. Chan C. K. (2010). Salicylaldehyde ester-induced chemoselective peptide ligations: enabling generation of natural peptidic linkages at the serine/threonine sites. Org. Lett. 12, 1724–1727. 10.1021/ol1003109 20232847

[B38] Liebscher S. Kornberger P. Fink G. Trost-Gross E. M. Höss E. Skerra A.. (2014a). Derivatization of antibody Fab fragments: a designer enzyme for native protein modification. ChemBioChem 15, 1096–1100. 10.1002/cbic.201400059 24782039

[B39] Liebscher S. Schöpfel M. Aumüller T. Sharkhuukhen A. Pech A. Höss E.. (2014b). N-terminal protein modification by substrate-activated reverse proteolysis. Angew. Chem. Int. Ed. 53, 3024–3028. 10.1002/anie.201307736 24520050

[B40] Ling J. J. Policarpo R. L. Rabideau A. E. Liao X. Pentelute B. L. (2012). Protein thioester synthesis enabled by sortase. J. Am. Chem. Soc. 134, 10749–10752. 10.1021/ja302354v 22686546 PMC3465687

[B41] Liu C.-F. Cao Y. Nguyen G. K. T. Tam J. P. (2015). Butelase-mediated synthesis of protein thioesters and its application for tandem chemoenzymatic ligation. Chem. Commun. 51, 17289–17292. 10.1039/C5CC07227A 26462854

[B42] Liu C. F. Tam J. P. (1994). Peptide segment ligation strategy without use of protecting groups. Proc. Natl. Acad. Sci. U.S.A. 91, 6584–6588. 10.1073/pnas.91.14.6584 8022823 PMC44247

[B43] Liu F. Luo E. Y. Flora D. B. Mezo A. R. (2014). A synthetic route to human insulin using isoacyl peptides. J. Org. Chem. 79, 487–492. 10.1021/jo4024914 24615765

[B44] Liu H. Li X. (2018). Serine/threonine ligation: origin, mechanistic aspects, and applications. Acc. Chem. Res. 51, 1643–1655. 10.1021/acs.accounts.8b00151 29979577

[B45] Maly D. J. Choong I. C. Ellman J. A. Saxon E. Bertozzi C. R. (2000). Traceless staudinger ligation for the chemoselective synthesis of amide bonds. Org. Lett. A. 2, 2141–2143. 10.1021/ol006054v 10891251

[B46] Mao H. Hart S. A. Schink A. Pollok B. A. (2004). Sortase A-mediated on-resin peptide cleavage and in situ ligation: an efficient one-pot strategy for the synthesis of functional peptides and proteins. J. Am. Chem. Soc. 126, 2670 −2671. 10.1021/ja039915e 14995162

[B47] Marraffini L. A. DeDent A. C. Schneewind O. (2006). Sortases and the art of anchoring proteins to the envelopes of gram-positive bacteria. Microbiol. Mol. Biol. Rev. 70, 192–221. 10.1128/MMBR.70.1.192-221.2006 16524923 PMC1393253

[B48] Matsumoto T. Isogawa Y. Minamihata K. Tanaka T. Kondo A. (2016). Twigged streptavidin polymer as a scaffold for protein assembly. J. Biotechnol. 225, 61–66. 10.1016/j.jbiotec.2016.03.030 27002233

[B49] Matsumoto T. Tanaka T. Kondo A. (2012). Sortase A-catalyzed site-specific coimmobilization on microparticles via streptavidin. Langmuir 28, 3553–3557. 10.1021/la2047933 22276782

[B50] Meyer C. Liebscher S. Bordusa F. (2016). Selective coupling of click anchors to proteins via trypsiligase. Bioconjug. Chem. 27, 47–53. 10.1021/acs.bioconjchem.5b00618 26670641

[B51] Nguyen G. K. T. Cao Y. Wang W. Liu C. F. Tam J. P. (2015a). Site-specific N-terminal labeling of peptides and proteins using butelase 1 and thiodepsipeptide. Angew. Chem. Int. Ed. 54, 15694–15698. 10.1002/anie.201506810 26563575

[B52] Nguyen G. K. T. Hemu X. Quek J.-P. Tam J. P. (2016a). Enzymatic engineering of live bacterial cell surface using butelase 1. Angew. Chemie Int. Ed. 55, 12802–12806. 10.1002/anie.201607188

[B53] Nguyen G. K. T. Kam A. Loo S. Jansson A. E. Pan L. X. Tam J. P. (2015b). Butelase 1: a versatile ligase for peptide and protein macrocyclization. J. Am. Chem. Soc. 137, 15398–15401. 10.1021/jacs.5b11014 26633100

[B54] Nguyen G. K. T. Qiu Y. Cao Y. Hemu X. Liu C. Tam J. P. (2016b). Butelase-mediated cyclization and ligation of peptides and proteins. Nat. Protoc. 11, 1977–1988. 10.1038/nprot.2016.118 27658013

[B55] Nguyen G. K. T. Wang S. Qiu Y. Hemu X. Lian Y. Tam J. P. (2014). Butelase 1 is an Asx-specific ligase enabling peptide macrocyclization and synthesis. Nat. Chem. Biol. 10, 732–738. 10.1038/nchembio.1586 25038786

[B56] Nikghalb K. D. Horvath N. M. Prelesnik J. L. Banks O. G. B. Filipov P. A. Row R. D.. (2018). Expanding the scope of sortase-mediated ligations by using sortase homologues. ChemBioChem 19, 185–195. 10.1002/cbic.201700517 29124839

[B57] Nilsson B. L. Kiessling L. L. Raines R. T. (2000). Staudinger ligation: a peptide from a thioester and azide. Org. Lett. 2, 1939–1941. 10.1021/ol0060174 10891196

[B58] Nuijens T. (2016). Chemo-Enzymatic Peptide Synthesis (CEPS): A Generally Applicable, Traceless Ligation Technology for the Synthesis of Peptide-to-Peptide and Peptide-to-Protein Conjugates. La Jolla, CA: Peptide Therapeutics Foundation.

[B59] Nuijens T. Quaedflieg P. J. L. M. Jakubke H. D. (2012). Hydrolysis and synthesis of peptides, in Enzyme Catalysis in Organic Synthesis, eds Drauz K. Gröger H. May O. (Heidelberg: WILEY-VCH Verlag), 675–748. 10.1002/9783527639861.ch17

[B60] Nuijens T. Schmidt M. (2019). Enzyme-Mediated Ligation Methods, Methods in Molecular Biology. Amsterdam: Springer Nature 10.1007/978-1-4939-9546-2

[B61] Nuijens T. Toplak A. Quaedflieg P. J. L. M. Drenth J. Wu B. Janssen D. B. (2016a). Engineering a diverse ligase toolbox for peptide segment condensation. Adv. Synth. Catal. 358, 4041–4048. 10.1002/adsc.201600774

[B62] Nuijens T. Toplak A. Van De Meulenreek M. B. A. C. Schmidt M. Goldbach M. Quaedflieg P. J. L. M. (2016b). Improved solid phase synthesis of peptide carboxyamidomethyl (Cam) esters for enzymatic segment condensation. Tetrahedron Lett. 57, 3635–3638. 10.1016/j.tetlet.2016.06.132

[B63] Nuijens T. Toplak A. van de Meulenreek M. B. A. C. Schmidt M. Goldbach M. Quaedflieg P. J. L. M. (2016c). Chemo-enzymatic peptide synthesis (CEPS) using omniligases and selective peptiligases Efficient biocatalysts for assembling linear and cyclic peptides and protein conjugates. Chim. Oggi Chem. Today. 34, 16–19.

[B64] Omura K. Aiba Y. Onoda H. Stanfield J. K. Ariyasu S. Sugimoto H.. (2018). Reconstitution of full-length P450BM3 with an artificial metal complex by utilising the transpeptidase Sortase A. Chem. Commun. 54, 7892–7895. 10.1039/C8CC02760A 29845154

[B65] Ott W. Nicolaus T. Gaub H. E. Nash M. A. (2016). Sequence-independent cloning and post-translational modification of repetitive protein polymers through sortase and Sfp-mediated enzymatic ligation. Biomacromolecules 17, 1330–1338. 10.1021/acs.biomac.5b01726 26974874

[B66] Parthasarathy R. Subramanian S. Boder E. T. (2007). Species- and cell type-specific interactions between CD47 and human SIRPalpha. Bioconjug. Chem. 18, 469–476. 10.1021/bc060339w 17302384

[B67] Pawlas J. Nuijens T. Persson J. Svensson T. Schmidt M. Toplak A. (2019). Sustainable, cost-efficient manufacturing of therapeutic peptides using chemo-enzymatic peptide synthesis (CEPS). Green Chem. 10.1039/C9GC03600H. [Epub ahead of print].

[B68] Pelay-Gimeno M. Bange T. Hennig S. Grossmann T. N. (2018). *In situ* cyclization of native proteins: structure-based design of a bicyclic enzyme. Angew. Chem. Int. Ed. 57, 11164–11170. 10.1002/anie.201804506 29847004 PMC6120448

[B69] Polgár L. Bender M. L. (1967). The reactivity of thiol-subtilisin, an enzyme containing a synthetic functional group. Biochemistry 6, 610–620. 10.1021/bi00854a032 6047645

[B70] Policarpo R. L. Kang H. Liao X. Rabideau A. E. Simon M. D. Pentelute B. L. (2014). Sortase-mediated chemical protein synthesis reveals the bidentate binding of bisphosphorylated p62 with K63 diubiquitin. Angew. Chemie Int. Ed. 53, 9203–9208. 10.1002/anie.201403582

[B71] Popp M. W. Dougan S. K. Chuang T.-Y. Spooner E. Ploegh H. L. Steiner D. F. (2011). Sortase-catalyzed transformations that improve the properties of cytokines. *Proc. Natl. Acad. Sci*. U.S.A. 108, 3169–3174. 10.1073/pnas.1016863108 PMC3044387 21297034

[B72] Pusterla I. Bode J. W. (2012). The mechanism of the α-ketoacid-hydroxylamine amide-forming ligation. Angew. Chem. Int. Ed. 51, 513–516. 10.1002/anie.201107198 22125261

[B73] Pusterla I. Bode J. W. (2015). An oxazetidine amino acid for chemical protein synthesis by rapid, serine-forming ligations. Nat. Chem. 7, 668–672. 10.1038/nchem.2282 26201744

[B74] Raltchev K. Pipercevic J. Hagn F. (2018). Production and structural analysis of membrane-anchored proteins in phospholipid nanodiscs. Chem. Eur. J. 24, 5493 −5499. 10.1002/chem.201800812 29457664

[B75] Rasche N. Tonillo J. Rieker M. Becker S. Dorr B. Ter-Ovanesyan D.. (2016). PROLink-single step circularization and purification procedure for the generation of an improved variant of human growth hormone. Bioconjug. Chem. 27, 1341–1347. 10.1021/acs.bioconjchem.6b00137 27108993

[B76] Richelle G. J. J. Schmidt M. Ippel H. Hackeng T. M. van Maarseveen J. H. Nuijens T.. (2018). A one-pot Triple-C multicyclization methodology for the synthesis of highly constrained isomerically pure tetracyclic peptides. ChemBioChem 19, 1934–1938. 10.1002/cbic.201800346 29944773

[B77] Rohde H. Seitz O. (2010). Ligation-desulfurization: a powerful combination in the synthesis of peptides and glycopeptides. Biopolymers 94, 551–559. 10.1002/bip.21442 20593472

[B78] Schmidt M. (2019). Enzymatic tools for peptide ligation and cyclization, Development and applications (dissertation). University of Amsterdam, Amsterdam, Netherlands.

[B79] Schmidt M. Huang Y.-H. de Oliveira E. F. T. Toplak A. Wijma H. J. Janssen D. B.. (2019). Efficient enzymatic cyclization of disulfide-rich peptides using peptide ligases. ChemBioChem. 20, 1524–1529. 10.1002/cbic.201900033 30735312

[B80] Schmidt M. Toplak A. Quaedflieg P. J. Nuijens T. (2017b). Enzyme-mediated ligation technologies for peptides and proteins. Curr. Opin. Chem. Biol. 38, 1–7. 10.1016/j.cbpa.2017.01.017 28229906

[B81] Schmidt M. Toplak A. Quaedflieg P. J. L. M. Ippel H. Richelle G. J. J. Hackeng T. M. (2017a). Omniligase-1: a powerful tool for peptide head-to-tail cyclization. Adv. Synth. Catal. 359, 2050–2055. 10.1002/adsc.201700314

[B82] Schmidt M. Toplak A. Rozeboom H. J. Wijma H. J. Quaedflieg P. J. L. M. Van Maarseveen J. H. (2018). Design of a substrate-tailored peptiligase variant for the efficient synthesis of thymosin-alpha(1). Org. Biomol. Chem. 16, 609–618. 10.1039/C7OB02812A 29300408

[B83] Sonntag M. Kumar P. Jagtap A. Simon B. Appavou M. Geerlof A.. (2017). Segmental, domain-selective perdeuteration and small-angle neutron scattering for structural analysis of multi-domain proteins. Angew. Chem. Int. Ed. 56, 9322–9325. 10.1002/anie.201702904 28636238

[B84] Stanger K. Maurer T. Kaluarachchi H. Coons M. Franke Y. Hannoush R. N.. (2014). Backbone cyclization of a recombinant cystine-knot peptide by engineered Sortase A. FEBS Lett. 588, 4487–4496. 10.1016/j.febslet.2014.10.020 25448598

[B85] Streefkerk D. E. Schmidt M. Ippel H. Hackeng T. M. Nuijens T. Timmermann P.. (2019). Synthesis of constrained tetracyclic peptides by consecutive CEPS, CLIPS, and oxime ligation. Org. Lett. 21, 2095–2100. 10.1021/acs.orglett.9b00378 30912446 PMC6456872

[B86] Swee L. K. Lourido S. Bell G. W. Ingram J. R. Ploegh H. L. (2015). One-step enzymatic modification of the cell surface redirects cellular cytotoxicity and parasite tropism. ACS Chem. Biol. 10, 460–465. 10.1021/cb500462t 25360987 PMC4478597

[B87] Ta D. T. Vanella R. Nash M. A. (2018). Bioorthogonal elastin-like polypeptide scaffolds for immunoassay enhancement. ACS Appl. Mater. Interfaces. 10, 30147–30154. 10.1021/acsami.8b10092 30125079

[B88] Tan X. Yang R. Liu C.-F. (2018). Facilitating subtiligase-catalyzed peptide ligation reactions by using peptide thioester substrates. Org. Lett. 20, 6691–6694. 10.1021/acs.orglett.8b02747 30350676

[B89] Toplak A. Nuijens T. Quaedflieg P. J. L. M. Wu B. Janssen D. B. (2016). Peptiligase, an enzyme for efficient chemoenzymatic peptide synthesis and cyclization in water. Adv. Synth. Catal. 358, 2140–2147. 10.1002/adsc.201600017

[B90] Touti F. Lautrette G. Johnson K. D. Delaney J. C. Wollacott A. Tissire H.. (2018). Antibody-bactericidal macrocyclic peptide conjugates to target gram-negative bacteria. Chem Bio Chem. 19, 2039–2044. 10.1002/cbic.201800295 29984452

[B91] Tung C. L. Wong C. T. Li X. (2015). Peptide 2-formylthiophenol esters do not proceed through a Ser/Thr ligation pathway, but participate in a peptide aminolysis to enable peptide condensation and cyclization. Org. Biomol. Chem. 13, 6922–6926. 10.1039/c5ob00825e 26013965

[B92] Van Lith S. A. M. Van Den Brand D. Wallbrecher R. (2017). A conjugate of an anti-epidermal growth factor receptor (EGFR) VHH and a cell-penetrating peptide drives receptor internalization and blocks EGFR activation. ChemBioChem 18, 2390–2394. 10.1002/cbic.201700444 28994180

[B93] van't Hof W. Hansenová Manásková S. Veerman E. C. Bolscher J. G. (2015). Sortase-mediated backbone cyclization of proteins and peptides. Biol. Chem. 396, 283–293. 10.1515/hsz-2014-0260 25581753

[B94] Wang H. H. Altun B. Nwe K. Tsourkas A. (2017). Proximity-based sortase-mediated ligation. Angew. Chem. Int. Ed. 56, 5349–5352. 10.1002/anie.201701419 28374553 PMC5537000

[B95] Wang J. H. Hu M. J. Zhang L. Shao X. X. Lv C. H. Liu Y. L.. (2018). Exploring receptor selectivity of the chimeric relaxin family peptide R3/I5 by incorporating unnatural amino acids. Biochimie 154, 77–85. 10.1016/j.biochi.2018.08.003 30102931

[B96] Weeks A. M. Wells J. A. (2017). Engineering peptide ligase specificity by proteomic identification of ligation sites. Nat. Chem. Biol. 14, 50–57. 10.1038/nchembio.2521 29155430 PMC5726896

[B97] Weeks A. M. Wells J. A. (2019). Subtiligase-catalyzed peptide ligation. Chem. Rev. 10.1021/acs.chemrev.9b00372. [Epub ahead of print]. 31663725

[B98] Williams F. P. Milbradt A. G. Embrey K. J. Bobby R. (2016). Segmental isotope labelling of an individual bromodomain of a tandem domain BRD4 using Sortase A. PLoS ONE. 11:e0154607. 10.1371/journal.pone.0154607 27128490 PMC4851411

[B99] Williamson D. J. Fascione M. A. Webb M. E. Turnbull W. B. (2012). Efficient N-terminal labeling of proteins by use of sortase. Angew. Chem. Int. Ed. 51, 9377–9380. 10.1002/anie.201204538 22890696

[B100] Williamson D. J. Webb M. E. Turnbull W. B. (2014). Depsipeptide substrates for sortase-mediated N-terminal protein ligation. Nat. Protoc. 9, 253–262. 10.1038/nprot.2014.003 24407354

[B101] Wu Z. Guo X. Zhongwu G. Guo Z. (2011). Sortase A-catalyzed peptidecyclization for the synthesis of macrocyclic peptides and glycopeptides. Chem. Commun. 47, 9218–9220. 10.1039/c1cc13322e 21738926 PMC3174090

[B102] Wu Z.-M. Liu S.-Z. Cheng X.-Z. Zhao X.-R. Hong H.-F. (2017). High yield synthesis of cyclic analogues of antibacterial peptides P-113 by Sortase A-mediated ligation and their conformation studies. Chinese Chem. Lett. 28, 553–557. 10.1016/j.cclet.2016.11.001

[B103] Yang R. Wong Y. H. Nguyen G. K. T. Tam J. P. Lescar J. Wu B. (2017). Engineering a catalytically efficient recombinant protein ligase. J. Am. Chem. Soc. 139, 5351–5358. 10.1021/jacs.6b12637 28199119

[B104] Zhang J. Yamaguchi S. Nagamune T. (2015). Sortase A-mediated synthesis of ligand-grafted cyclized peptides for modulating a model protein-protein interaction. Biotechnol. J. 10, 1499–1505. 10.1002/biot.201500013 25913771

[B105] Zhang Y. Xu C. Lam Y. Lee C. L. Li X. (2013). PNAS Protein chemical synthesis by serine and threonine ligation. *Proc. Natl. Acad. Sci*. U.S.A. 110, 6657–6662. 10.1073/pnas.1221012110 PMC3637748 23569249

